# Anti-proliferation of triple-negative breast cancer cells with physagulide P: ROS/JNK signaling pathway induces apoptosis and autophagic cell death

**DOI:** 10.18632/oncotarget.19299

**Published:** 2017-07-17

**Authors:** Pei Yu, Chao Zhang, Cai-Yun Gao, Ting Ma, Hao Zhang, Miao-Miao Zhou, Yan-Wei Yang, Lei Yang, Ling-Yi Kong

**Affiliations:** ^1^ Jiangsu Key Laboratory of Bioactive Natural Product Research and State Key Laboratory of Natural Medicines, China Pharmaceutical University, Nanjing 210009, China

**Keywords:** physagulide P, cell cycle arrest, apoptosis, autophagy, ROS/JNK

## Abstract

Physagulide P (PP), a new natural compound, was isolated from *Physalis angulate L. i*n our laboratory. In this study, we demonstrated that PP potently suppressed cell proliferation by inducing G2/M phase arrest in MDA-MB-231 and MDA-MB-468 cells. Moreover, PP provoked apoptosis by decreasing the mitochondrial membrane potential and elevating the Bax/Bcl-2 protein expression ratio. The caspase inhibitor Z-VAD-FMK partly restore cell viability, suggesting that apoptosis plays as an important role in the anti-proliferative effect of PP. PP-treated cells also underwent autophagy, as evidenced by the formation of autophagosomes and the accumulation of LC3BII. Furthermore, the knockdown of LC3B reduced PP-induced cytotoxicity, indicating that autophagy played an anticancer effect. PP also induced the generation of reactive oxygen species (ROS) and resulted in c-Jun N-terminal kinases (JNK) activation. Accordingly, JNK siRNA significantly attenuated PP-triggered apoptosis and autophagy, and ROS scavengers almost completely reverse this apoptosis and autophagy. The ROS scavenger also blocked PP-induced G2/M phase arrest and the phosphorylation of JNK. Our results revealed that PP induced G2/M phase arrest, apoptosis and autophagy via the ROS/JNK signaling pathway in MDA-MB-231 and MDA-MB-468 cells. Therefore, PP is a promising candidate for the development of antitumor drugs for the treatment of triple-negative breast cancer.

## INTRODUCTION

Breast cancer is the most common cancer among women worldwide [1]. In the United States, 231,840 new cases of invasive breast cancer and 40,290 deaths from this disease are estimated to occur in 2015, and one in eight women will develop breast cancer during their lifetime [2]. Triple-negative breast cancer, defined by the lack of expression of estrogen receptorα (ERα) and progesterone receptor (PR) as well as the absence of overexpression and/or gene amplification of HER2, is a unique subtype of breast cancer with limited treatment options and poor prognosis [3, 4]. Compared with any other subtypes of breast cancer, triple-negative breast cancer follows a more aggressive clinical course and shows a higher risk of recurrence and death in the first 3 to 5 years after diagnosis [5]. In addition, current treatment regimens lead to chemoresistance and toxicity [6]. Hence, the development of novel therapeutic strategies as well as effective and less toxic drugs are essential in fighting triple-negative breast cancer.

Cell proliferation requires the completion of the cell cycle, which is regulated by cyclin-dependent kinases (CDKs) and CDK inhibitor proteins [7]. Specifically, a successful G2/M transition is important for cells proliferation, and this transition is regulated by cyclin family members, including Cyclin B1, Cdc2, Cdc25C, Chk1/2 and p21 [8–11]. Cell cycle regulation imbalance is a characteristic of cancer cells and promotes the occurrence and development of tumors [12].

Apoptosis, or type-I programmed cell death (PCD), involves the activation of catabolic enzymes – in particular proteases – in signaling cascades, which leads to cell membrane blebbing, cell shrinkage, nuclear fragmentation, chromatin condensation, DNA fragmentation, and the formation of apoptotic bodies [13–15]. Autophagy, which is classified as the type-II PCD, is a catabolic process that sequesters cytoplasmic proteins and organelles into autophagosomes and transports them to lysosomes for recycling and degradation [16, 17]. However, the ability of PP to induce apoptosis or autophagy as well as their roles and interplay in the PP-induced cell growth inhibition of triple-negative breast cancer cells remain to be determined.

ROS are mainly formed in mitochondria and are involved in the regulation of different physiological processes, including apoptosis and autophagy [18, 19]. ROS can affect various signaling pathways such as mitogen-activated protein kinase (MAPK) signal transduction cascades [20, 21]. Moreover, JNK, a stress-activated protein kinase (SAPK) of the MAPK family, plays a pivotal role in many cellular events, including apoptosis and autophagy [22, 23]. In short, targeting the ROS/JNK signaling pathway may be effective for the treatment of triple-negative breast cancer.

**Figure 1:**
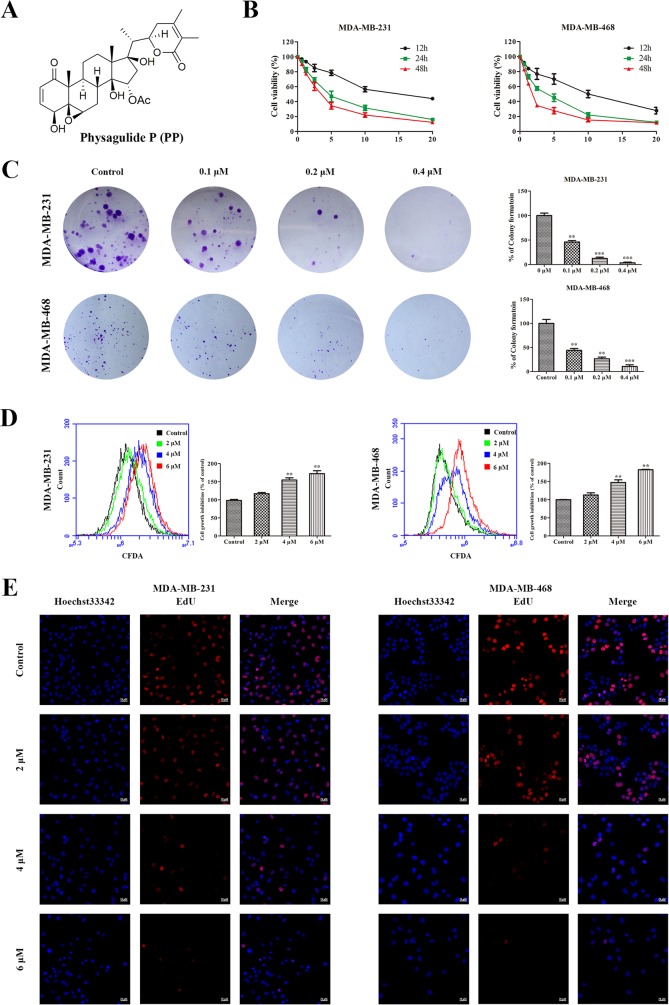
PP inhibited cell growth in triple-negative breast cancer cells **(A)** The chemical structure of physagulide P. **(B)** MDA-MB-231 and MDA-MB-468 cells were treated with different concentrations of PP for 12-48 h, and the cell viability was measured with an MTT assay. **(C)** MDA-MB-231 and MDA-MB-468 cells were treated with different concentrations of PP for 14 d and colony formation was assessed by staining with crystal violet. **(D)** MDA-MB-231 and MDA-MB-468 cells were treated with different concentrations of PP for 24 h, and cell division was assessed with a CFDA-SE assay. **(E)** MDA-MB-231 and MDA-MB-468 cells were incubated with various concentrations of PP for 24 h, stained with EdU and observed using a confocal microscopy. Scale bars = 20 μm. The results were similar in at least three independent experiments. *p < 0.05, **p < 0.01, vs. control group.

*Physalis angulata L*., a traditional Chinese herbal medicine, is an annual herb distributed in many countries located in tropical and subtropical regions of the world and is used worldwide for its fruits. Extracts or infusions from this plant have been used in various countries in popular medicine as a treatment for a variety of illnesses, such as malaria, asthma, hepatitis, dermatitis, rheumatism, wound healing, sleeping sickness, earache, and fever, among others [24–28]. In the present study, we report the novel anticancer effect of PP (Figure 1A) purified from *Physalis angulata L*. in triple-negative breast cancer. We further explored the molecular mechanisms, that is, the induction of G2/M phase arrest, apoptosis and autophagy mediated by the ROS/JNK signaling pathway.

## RESULTS

### PP inhibited the proliferation of triple-negative breast cancer cells

To investigate the anti-proliferative effect of PP on triple-negative breast cancer cells, MDA-MB-231 and MDA-MB-468 cells were treated with various concentrations of PP. 3-[4, 5-dimethylthiazole-2-yl]-2, 5-diphenyltetrazolium bromide (MTT) results showed that PP inhibited MDA-MB-231 and MDA-MB-468 cell growth in a dose- and time-dependent manner (Figure 1B). Moreover, a colony formation assay showed that fewer colonies formed after PP treatment, and this effect depended on dose (Figure 1C). MDA-MB-231 and MDA-MB-468 cells were also labeled with carboxyfluorescein diacetate, succinimidyl ester (CFDA-SE) to monitor the effect of PP on cell division. After entering the cell, CFDA-SE is equally distributed between the daughter cells when the cell divides; thus, its fluorescence intensity decreases as the cell divides. As shown in Figure 1D, the CFDA-SE fluorescence intensity increased in a dose-dependent manner after treatment with PP, suggesting that PP inhibited cell division and proliferation. To further confirm the effect of PP on cell proliferation, a 5-ethynyl-20-deoxyuridine (EdU) incorporation assay was employed, Figure 1E showes that PP significantly and dose-dependently reduced the number of EdU-positive cells compared with the control group. These results show that PP inhibits the proliferation of MDA-MB-231 and MDA-MB-468 cells in a dose- and time-dependent manner.

### PP induced G2/M phase arrest in triple-negative breast cancer cells

**Figure 2:**
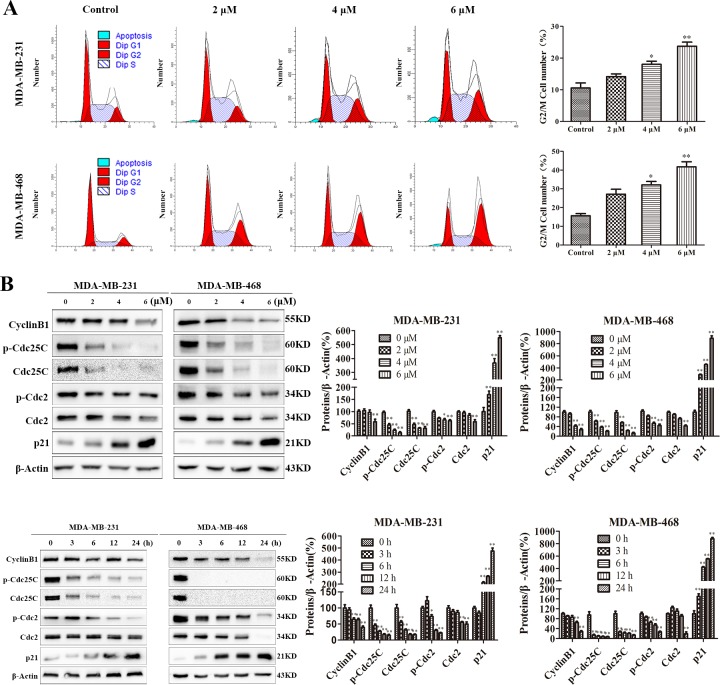
PP induced G2/M phase arrest in triple-negative breast cancer cells **(A)** MDA-MB-231 and MDA-MB-468 cells were treated with different concentrations of PP for 24 h, and the cell cycle distribution was measured using flow cytometry. **(B)** MDA-MB-231 and MDA-MB-468 cells were treated with different concentrations of PP for 24 h and 6 μM PP for different periods, and the expression levels of cell cycle-regulated proteins were measured by Western blotting. The results were similar in at least three independent experiments. *p < 0.05, **p < 0.01, vs. control group.

Cell cycle arrest inhibits cell proliferation. To investigate the role of cell cycle arrest in the PP-mediated inhibition of cell proliferation, we examined the distribution of cell cycle in cells treated with PP by flow cytometry after staining with PI. As shown in Figure 2A, PP led to the accumulation of cells in the G2/M phase in a dose-dependent manner. To elucidate the mechanisms underlying this effect, we measured the expression levels of cell cycle-regulated proteins. A Western blotting analysis showed that PP up-regulated the expressions of p21, and down-regulated the levels of Cyclin B1 phospho-Cdc25C, Cdc25C, phospho-Cdc2 and Cdc2 (Figure 2B). Taken together, these data suggest that the PP may alter the expression of cell-cycle related proteins to induce G2/M phase arrest and consequently inhibit the proliferation of triple-negative breast cancer cells.

### PP triggered mitochondrial apoptosis in triple-negative breast cancer cells

**Figure 3:**
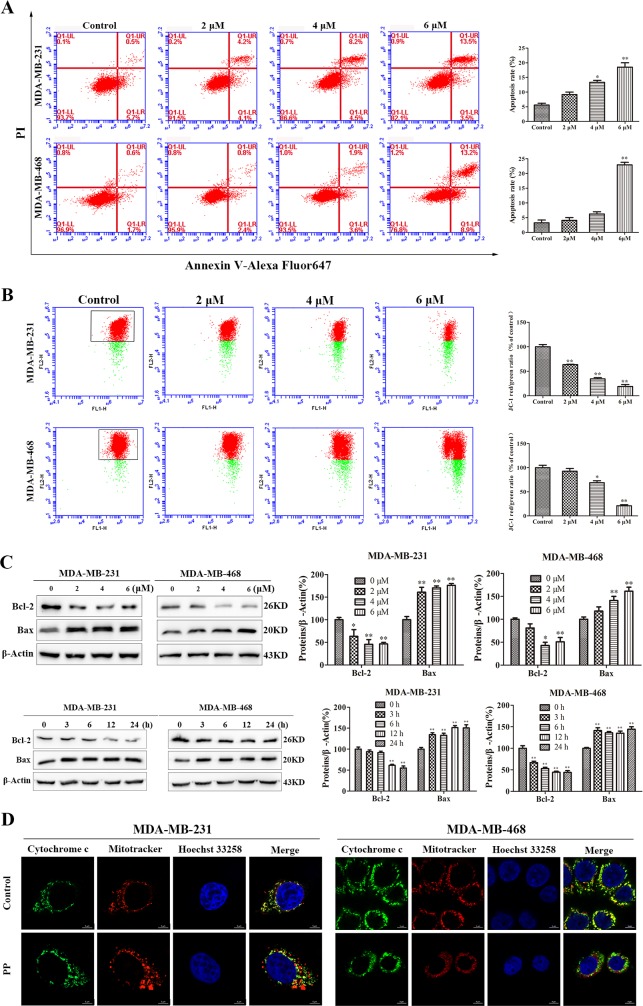
PP induced mitochondrial dysfunction in triple-negative breast cancer cells **(A)** The rates of apoptotic MDA-MB-231 and MDA-MB-468 cells after treatment with PP for 24 h, as determined by Annexin V-Alexa Fluor 647 and PI staining. **(B)** The mitochondrial membrane potential of MDA-MB-231 and MDA-MB-468 cells treated with PP for 24 h, as measured by flow cytometry with JC-1 staining. **(C)** The expressions of Bax and Bcl-2 in MDA-MB-231 and MDA-MB-468 cells after treatment with various concentrations of PP for 24 h and 6 μM PP for different periods. **(D)** MDA-MB-231 and MDA-MB-468 cells were treated with 6 μM PP for 24 h, and their immunofluorescence was assessed. Green: FITC-labeled Cytochrome c; Red: Mito-Tracker-labeled mitochondria; Blue: Hoechst 33258-labeled nuclei. Scale bars = 5 μm. The results were similar in at least three independent experiments. *p < 0.05, **p < 0.01, vs. control group.

To examine whether the cell growth inhibition induced by PP also depends on apoptosis, PP-treated cells were stained with Annexin V-Alexa Fluor 647/propidium iodide (PI), which showed that PP treatment induced remarkable apoptosis comparing to the control group (Figure 3A). We then measured the mitochondrial membrane potential (Δ*Ψ_m_*) by flow cytometry and found that PP treatment decreased the Δ*Ψ_m_* in a dose-dependent manner (Figure 3B). In addition to this change in Δ*Ψ_m_*, increased Bax and decreased Bcl-2 protein expression were also observed after treatment with PP (Figure 3C). Δ*Ψ_m_* is well known to play an important role in the release of Cytochrome c (Cyt c). Thus, Cyt c expression was further investigated by immunofluorescence. As shown in Figure 3D, Cyt c localizes to the inner mitochondrial membrane of untreated cells, but it was released into the cytosol after treatment with PP for 24 h. These results demonstrated that PP triggered apoptosis by inducing mitochondrial membrane depolarization and Cyt c release.

**Figure 4:**
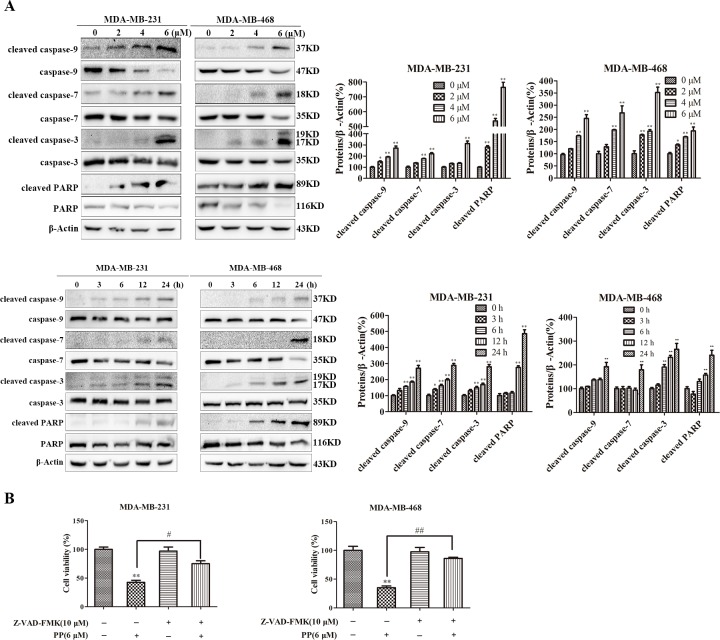
PP induced apoptosis in triple-negative breast cancer cells **(A)** The expression of cleaved caspase-9, caspase-9, cleaved caspase-7, caspase-7, cleaved caspase-3, caspase-3, cleaved PARP and PARP in MDA-MB-231 and MDA-MB-468 cells after treatment with various concentrations of PP for 24 h and 6 μM PP for different periods. **(B)** MDA-MB-231 and MDA-MB-468 cells were treated with 6 μM PP alone or in combination with the pan-caspase inhibitor Z-VAD-FMK (10 μM) for 24 h, and then cell viability was assessed with an MTT assay. The results were similar in at least three independent experiments. *p < 0.05, **p < 0.01, vs. control group; ^#^*p* < 0.05, ^##^*p* < 0.01 vs. PP group.

In the absence of functional mitochondria, apopto-somes form and activate the apoptosis process. In our study, we observed significant caspase-9, caspase-7, caspase-3 and PARP processing in total cell lysates from PP-treated cells, and the cleavage of caspase-9, caspase-7, caspase-3 and PARP markedly increased in both a time- and dose-dependent manner in MDA-MB-231 and MDA-MB-468 cells (Figure 4A). Furthermore, when MDA-MB-231 and MDA-MB-468 cells were pre-treated with the pan-caspase inhibitor Z-VAD-FMK (10 μM) for 1 h before treatment with PP, cell viability was partly restored (Figure 4B). Taken together, these data suggest that the cytotoxic effects of PP on MDA-MB-231 and MDA-MB-468 cells were partly caused by the activation of caspase-dependent apoptosis.

### PP induced autophagic cell growth inhibition in triple-negative breast cancer cells

**Figure 5:**
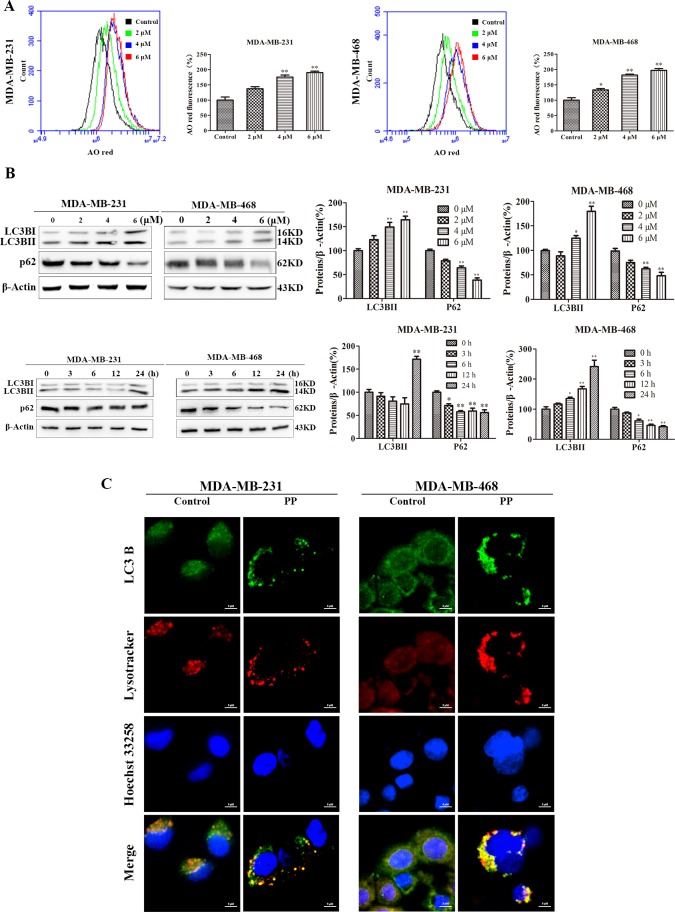
PP promoted the formation of autophagosome in triple-negative breast cancer cells **(A)** MDA-MB-231 and MDA-MB-468 cells were treated with different concentrations of PP for 24 h, stained with AO and analyzed by flow cytometry. **(B)** Expression levels of LC3BII and p62 by Western blotting in MDA-MB-231 and MDA-MB-468 cells treated with different concentrations of PP and 6 μM PP for different periods. **(C)** MDA-MB-231 and MDA-MB-468 cells were treated with 6 μM PP for 18 h ad analyzed by immunofluorescence. Green: FITC-labeled LC3B; Red: Lyso-Tracker-labeled lysosomes; Blue: Hoechst 33258-labeled nuclei. Scale bars = 5 μm. The results were similar in at least three independent experiments. *p < 0.05, **p < 0.01, vs. control group.

**Figure 6:**
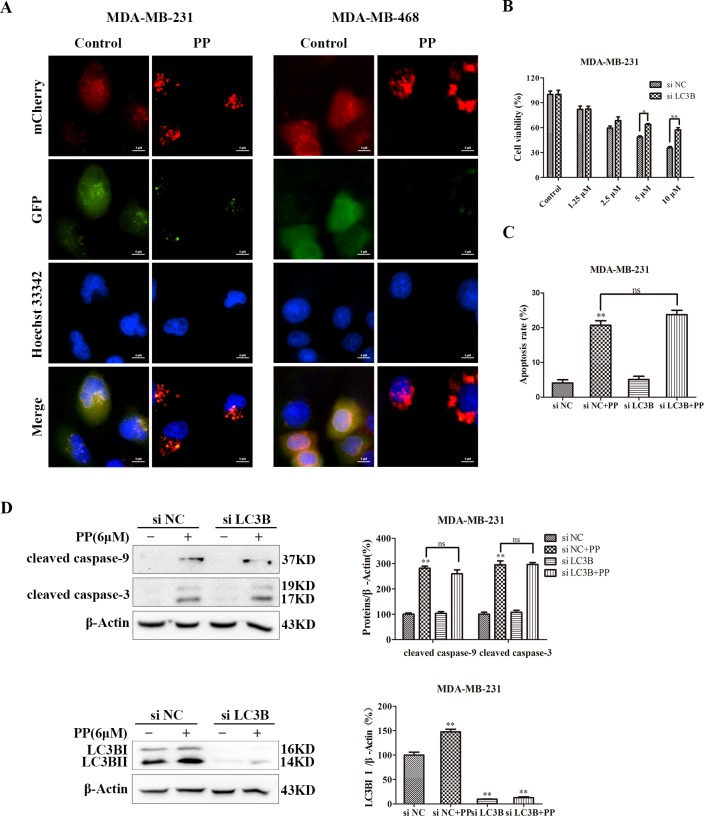
PP induced autophagic cell growth inhibition in triple-negative breast cancer cells **(A)** Representative images of MDA-MB-231 and MDA-MB-468 cells transfected with Ad-mCherry-GFP-LC3B adenovirus after PP treatment (6 μM, 18 h). Scale bars = 5 μm. **(B)** MDA-MB-231 cells were transfected with 100 nM of LC3B siRNA for 24 h and then treated with different concentrations of PP for 24 h. The viability of MDA-MB-231 cells was then assessed with an MTT assay. **(C)** Apoptotic MDA-MB-231 cells were detected by flow cytometry after treatment with 6 μM PP for 24 h in the presence or absence of LC3B siRNA. **(D)** The protein expression levels of cleaved caspase-9, cleaved caspase-3 and LC3BII in MDA-MB-231 cells transfected with LC3B siRNA in the presence or absence of 6 μM PP. The results were similar in at least three independent experiments. *p < 0.05, **p < 0.01 vs. si NC group.

Autophagy, i.e., type-II PCD, plays a key role in the antiproliferative effects of external factors. As shown in Figure 5A, the concentration-dependent formation of acidic vesicular organelles (AVOs) was evident in PP-treated cells stained with the lysosomo-tropic agent acridine orange. In addition, LC3BII expression was significantly increased and p62/SQSTM1 expression was significantly attenuated in a concentration- and time-dependent manner in both PP-treated cell lines (Figure 5B). Consistent with the results of the Western blotting analysis, the PP-treated MDA-MB-231 and MDA-MB-468 cells exhibited an increase in the punctate pattern of LC3B and lysosome fluorescence, which represent the recruitment of LC3B into autophagosomes and the formation of autophagy lysosomes (Figure 5C). To further demonstrate the formation of autophagy lysosomes, MDA-MB-231 and MDA-MB-468 cells were transfected with Ad-mCherry-GFP-LC3B adenovirus. Green fluorescence was almost completely quenched and appeared as red dots in PP-treated cells (Figure 6A), indicating that PP induced the formation of autophagy lysosomes. To determine the role of autophagosome accumulation in PP-induced cytotoxicity, we knocked down LC3B, which showed that the viability of the LC3B siRNA-treated group was higher than that of the NC siRNA group (Figure 6B). However, the apoptosis and the expression of cleaved caspase-9 and cleaved caspase-3 did not significantly differ between the LC3B siRNA group and the NC siRNA group (Figure 6C–6D). These results suggest that autophagy acts as another way participated in triple-negative breast cancer cell growth inhibition of PP.

### ROS mediated PP-induced G2/M arrest, apoptosis and autophagy in triple-negative breast cancer cells

**Figure 7:**
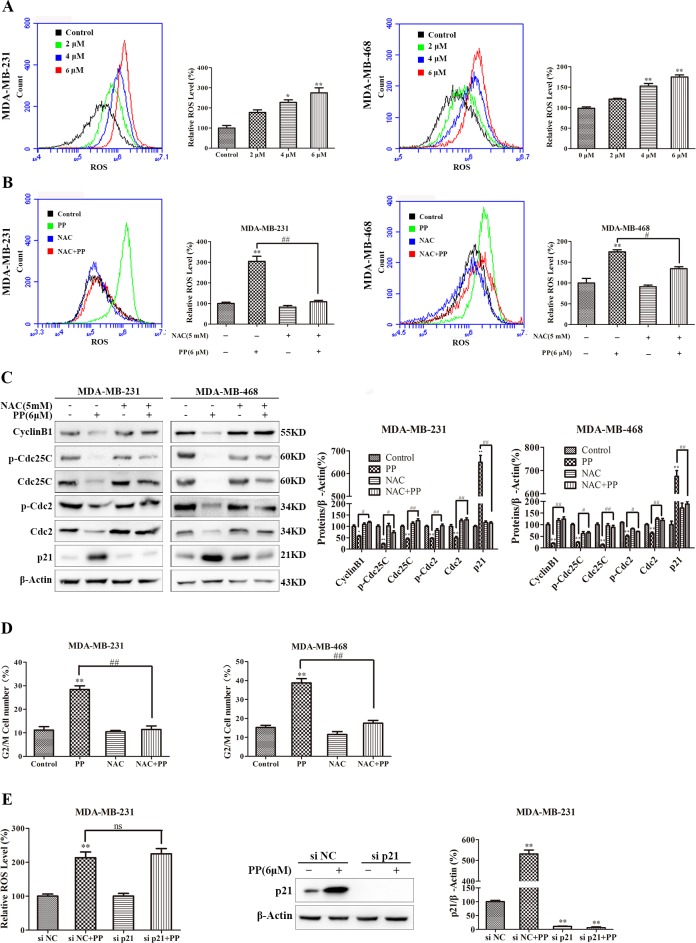
ROS mediated PP-induced G2/M arrest in triple-negative breast cancer cells **(A)** The ROS levels in MDA-MB-231 and MDA-MB-468 cells incubated with various concentrations of PP for 24 h were measured by flow cytometry. **(B)** The cells were treated with 6 μM PP alone or in combination with NAC (5 mM) and then subjected to flow cytometry to measure ROS levels. **(C)** The expression levels of cell cycle-regulated proteins in cells treated with 6 μM PP alone or in combination with NAC were measured by Western blotting. **(D)** The cell cycle was evaluated by flow cytometry in cells treated with 6 μM PP alone or in combination with NAC. **(E)** The ROS levels and the protein expression level of p21 in MDA-MB-231 cells transfected with p21 siRNA in the presence or absence of 6 μM PP. The results were similar in at least three independent experiments. *p < 0.05, **p < 0.01, vs. control group or si NC group; ^#^*p* < 0.05, ^##^*p* < 0.01 vs. PP group.

**Figure 8:**
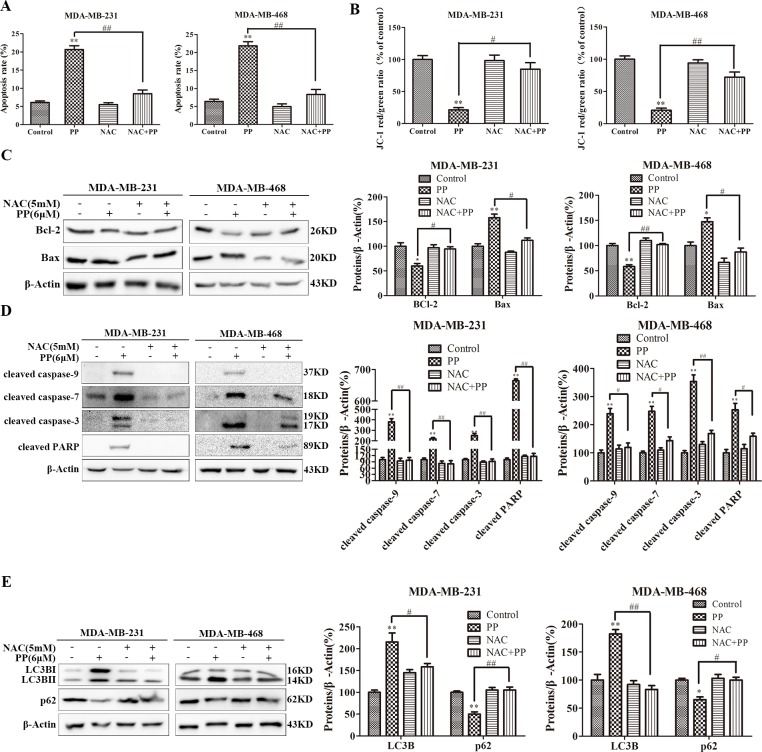
ROS mediated PP-induced autophagy and apoptosis in triple-negative breast cancer cells **(A)** Apoptosis was measured by flow cytometry with Annexin V- Alexa Fluor 647 and PI staining in cells treated with 6 μM PP alone or in combination with NAC. **(B)** The changes in mitochondrial membrane potential were assessed by flow cytometry in cells treated with 6 μM PP alone or in combination with NAC. **(C)** The protein expression levels of Bcl-2 and Bax in MDA-MB-231 and MDA-MB-468 cells treated with PP alone or in combination with NAC. **(D)** The proteins levels of cleaved caspase-9 and cleaved caspase-7, cleaved caspase-3, and cleaved PARP were measured in MDA-MB-231 and MDA-MB-468 cells treated with PP alone or in combination with NAC. **(E)** The protein expression levels of LC3BII and p62 were measured in MDA-MB-231 and MDA-MB-468 cells treated with PP alone or in combination with NAC. The results were similar in at least three independent experiments. *p < 0.05, **p < 0.01, vs. control group; ^#^*p* < 0.05, ^##^*p* < 0.01 vs. PP group.

ROS normally exists in all aerobic cells in balance with biochemical antioxidants, and this balance can determine the fate of cancer cells by regulating various signaling pathways, including cell cycle arrest, apoptosis, autophagy and necrosis. As shown in Figure 7A, the ROS level increased in MDA-MB-231 and MDA-MB-468 cells after treatment with different concentrations of PP for 24 h. Next, we applied NAC, a ROS inhibitor, to examine the role of ROS in PP-induced cytotoxicity in MDA-MB-231 and MDA-MB-468 cells. As expected, NAC strongly inhibited PP induced ROS generation (Figure 7B), and NAC also strongly inhibited PP-induced G2/M phase arrest and reversed changes in the expression of G2/M cell cycle regulator proteins (Figure 7C–7D). However, flow cytometry indicated that p21 siRNA failed to restore PP induced ROS generation (Figure 7E). Furthermore, the number of apoptotic cells decreased when MDA-MB-231 and MDA-MB-468 cells were treated with NAC and PP compared with the PP-treated group (Figure 8A). NAC also reversed the PP-induced decrease in the Δ*Ψ_m_*. (Figure 8B). Furthermore, the changes in cleaved caspase-9, cleaved caspase-7, cleaved caspase-3, cleaved PARP, Bcl-2 and Bax expression were also reversed when MDA-MB-231 and MDA-MB-468 cells were co-incubated with PP and NAC (Figure 8C–8D). With respect to autophagy makers, LC3BII expression decreased and p62 expression increased after treatment with PP and NAC compared with the PP-treated group (Figure 8E). Taken together, these results indicate that PP induces G2/M arrest, autophagy and apoptosis through upregulating ROS in triple-negative breast cancer cells.

### PP induced JNK activation dependent on ROS production

**Figure 9:**
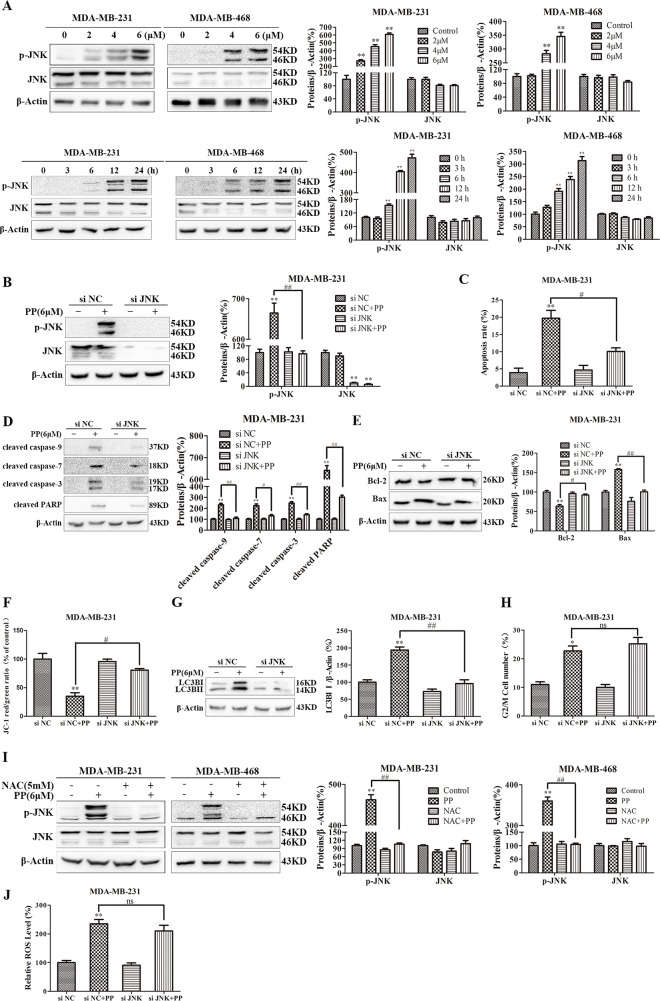
ROS-mediated JNK activation was crucial mediators of autophagy and apoptosis induced by PP in triple-negative breast cancer cells **(A)** MDA-MB-231 and MDA-MB-468 cells were treated with different concentrations of PP for 24 h and 6 μM PP for different periods, and the expression levels of JNK and phospho-JNK were then measured by Western blotting. **(B)** The protein expression levels of JNK and phospho-JNK in MDA-MB-231 cells transfected with JNK siRNA in the presence or absence of 6 μM PP. **(C)** Apoptotic MDA-MB-231 cells were measured by flow cytometry after treatment with 6 μM PP for 24 h in the presence or absence of JNK siRNA. **(D)** The protein levels of cleaved caspase-9, cleaved caspase-7, cleaved caspase-3 and cleaved PARP in MDA-MB-231 cells transfected with JNK siRNA in the presence or absence of 6 μM PP. **(E)** The protein expression levels of Bax and Bcl-2 in MDA-MB-231 cells transfected with JNK siRNA in the presence or absence of 6 μM PP. **(F)** The changes in mitochondrial membrane potential in MDA-MB-231 cells were assessed by flow cytometry after treatment with 6 μM PP for 24 h in the presence or absence of JNK siRNA. **(G)** The protein expression levels of LC3BII in MDA-MB-231 cells transfected with JNK siRNA in the presence or absence of 6 μM PP. **(H)** The cell cycle was assessed by flow cytometry in MDA-MB-231 cells after treatment with 6 μM PP for 24 h in the presence or absence of JNK siRNA. **(I)** The protein levels of JNK and phospho-JNK in MDA-MB-231 and MDA-MB-468 cells treated with PP alone or in combination with NAC. **(J)** The ROS levels in MDA-MB-231 cells were detected by flow cytometry after treatment with 6 μM PP for 24 h in the presence or absence of JNK siRNA. The results were similar in at least three independent experiments. *p < 0.05, **p < 0.01, vs. control group or si NC group; ^#^*p* < 0.05, ^##^*p* < 0.01 vs. PP/si NC+PP group.

We also investigated the effect of PP on JNK activation, and Figure 9A shows that PP increased the level of JNK phosphorylation in both MDA-MB-231 and MDA-MB-468 cells. We then used a specific JNK siRNA to assess the contribution of activated JNK to PP-induced apoptosis, autophagy or cell cycle arrest and found that the ablation of JNK with siRNA downregulated phospho-JNK in MDA-MB-231 cells, irrespective of PP treatment (Figure 9B). In addition, the number of PP-triggered apoptotic cells decreased when cells were transfected with JNK siRNA compared to the NC siRNA group (Figure 9C). When MDA-MB-231 cells were transfected with JNK siRNA and co-treated with PP, JNK siRNA blocked PP-induced caspase activation (Figure 9D). Similarly, JNK siRNA also blocked the PP-induced decrease in Δ*Ψ_m_* and the Bax/Bcl-2 ratio (Figure 9E–9F). Moreover, we found that the ablation of JNK decreased the effected of PP on LC3BII (Figure 9G). However, flow cytometry indicated that JNK siRNA failed to restore the PP-induced increase in G2/M phase arrest (Figure 9H). These results suggest that the activation of JNK is required for PP-induced apoptosis and autophagy but not involved in G2/M phase arrest. Subsequently, the relationship between ROS generation and JNK activation in PP-induced apoptosis and autophagy was investigated by treating of MDA-MB-231 and MDA-MB-468 cells with PP for 24 h alone or in combination with NAC. Changes in phospho-JNK expression were completely reversed when triple-negative breast cancer cells were treated with NAC (Figure 9I). In addition, JNK siRNA failed to restore PP induced ROS generation (Figure 9J). These results reveal that ROS is the proximal event of JNK and most likely initiates PP-induced apoptosis and autophagy. Thus, PP-induced ROS generation activates JNK signaling to inhibit the growth of triple-negative breast cancer cells.

## DISCUSSION

PP is a novel withanolide compound that was isolated from *Physalis angulate L* at our laboratory, and its analogues have been reported to be cytotoxic to many types of cancer cells via diverse mechanisms [29, 30]. In this study, we investigated the effect of PP on MDA-MB-231 and MDA-MB-468 cells. Our results confirm that PP inhibits proliferation of triple-negative breast cancer cells via G2/M arrest, apoptosis and autophagy mediated by ROS/JNK signaling.

Cell cycle deregulation is a hallmark of cancer, and the induction of cell cycle arrest, especially G2/M phase arrest, may be an effective strategy to treat aberrant cancer cell proliferation [31–33]. The Cyclin B1 and Cdc2 complex, which is rendered inactive by the phosphorylation of Cdc2, plays a key role in promoting the G2/M phase transition [11]. At the initiation of mitosis, cell division cycle protein (Cdc25C) is activated to dephosphorylate of Cdc2 [34, 35], and Cdc25C is phosphorylated by the checkpoint kinases Chk1 and Chk2 and downregulates Cdc25C activity [36, 37]. In addition, p21 plays a critical role in the G2/M checkpoint by inhibiting the Cdc2/cyclinB1 complex [38]. According to our flow cytometric analysis, PP treatment resulted in G2/M phase arrest in triple-negative breast cancer cells. Further Western blotting analyses showed that PP caused G2/M phase arrest by increasing the protein levels of p21 while decreasing the protein levels of Cdc2, phospho-Cdc2, phospho-Cdc25C, Cdc25C and CyclinB1.

Apoptosis is an evolutionarily conserved cell suicide mechanism to eliminate redundant, damaged, or infected cells [39] and can be activated by compounds targeting extrinsic and intrinsic apoptosis pathways [40]. The extrinsic apoptotic pathway involves the interaction of ligands with their respective cell surface death receptors, such as Fas/CD95 and TNFR1, which results in the activation of the proteases caspase-9, caspase-3 and other downstream caspases and the initiation of the apoptotic cascade [41]. Alternatively, the mitochondria-mediated intrinsic pathway may trigger apoptosis. Our results showed that Δ*Ψ_m_* sharply decreased after PP treatment, indicating that PP depolarized mitochondria in MDA-MB-231 and MDA-MB-468 cells. Mitochondrial membrane depolarization can release Cyt c from the mitochondrial intramembrane space to the cytosol and activate cytosolic caspases [42–44].

Autophagy, another caspase-independent anti-proliferation pathway, plays a significant role in determining cellular fate [45]. We found that PP induced autophagy, as evidenced by the accumulation of AO-staining acidic vesicles, the formation of autophagy lysosomes observed with an imageXpress® confocal microscope and the upregulation of LC3BII. To further explore the relationship between autophagy and cell growth inhibition, we transfected MDA-MB-231 cells with LC3B siRNA, which showed that LC3B knockdown markedly attenuated PP-mediated decreases in cell vitality, indicating that autophagy plays an anti-proliferative role. However, the LC3B siRNA-mediated decrease in autophagy did not affect the number of apoptotic cells and protein levels of cleaved caspase-9 and cleaved caspase-3, which indicates that autophagy and apoptosis independently inhibit cell growth.

The generation of ROS in MDA-MB-231 and MDA-MB-468 cells is a striking feature of PP-induced apoptosis and autophagy. Specifically, ROS are important signaling molecules that regulate many signal-transduction pathways [46–48], and excessive ROS generation may interfere with cellular signaling pathways and play critical roles in inducing both cell apoptosis and autophagy [49, 50]. Furthermore, emerging evidences illustrated that natural compounds such as Physalin A [51], withaferin A [27] and celastrol [11] can cause ROS-mediated apoptosis and autophagy due to their structure of α, β-unsaturated ketone moieties [52]. PP also has an α, β-unsaturated ketone moiety, thus we hypothesized that PP-induced apoptosis and autophagy may be mediated by ROS. In this study, PP significantly increased ROS generation in MDA-MB-231 and MDA-MB-468 cells, and the ROS inhibitor NAC reversed the PP-induced inhibition of cell proliferation. NAC blocked PP-induced G2/M arrest, apoptosis and autophagy in MDA-MB-231 and MDA-MB-468 cells, suggesting that PP affected cell proliferation via the ROS-mediated modulation of the cell cycle, apoptosis and autophagy.

JNK, a member of the MAPK family, transduces cellular signals in response to different physiological stimuli and stresses [49]. To explore the relationship between ROS and JNK as well as their role in G2/M phase arrest, the ability of PP to activate JNK was investigated. PP induced ROS production in MDA-MB-231 and MDA-MB-468, which strongly activated JNK (phosphorylation). Moreover, NAC almost completely reversed the PP-induced inhibition of apoptosis and autophagy, and JNK siRNA significantly attenuated these two processes. NAC, but not JNK siRNA, also strongly blocked G2/M phase arrest, indicating that ROS, but not JNK, mediated PP-induced cell cycle arrest. In addition, NAC completely blocked JNK phosphorylation, implying that ROS is the proximal event of JNK signaling. In summary, our studies provide experimental evidence to support that PP induces apoptosis and autophagy via the ROS/JNK signaling pathway and that ROS play a vital role in G2/M phase arrest caused by PP.

**Figure 10:**
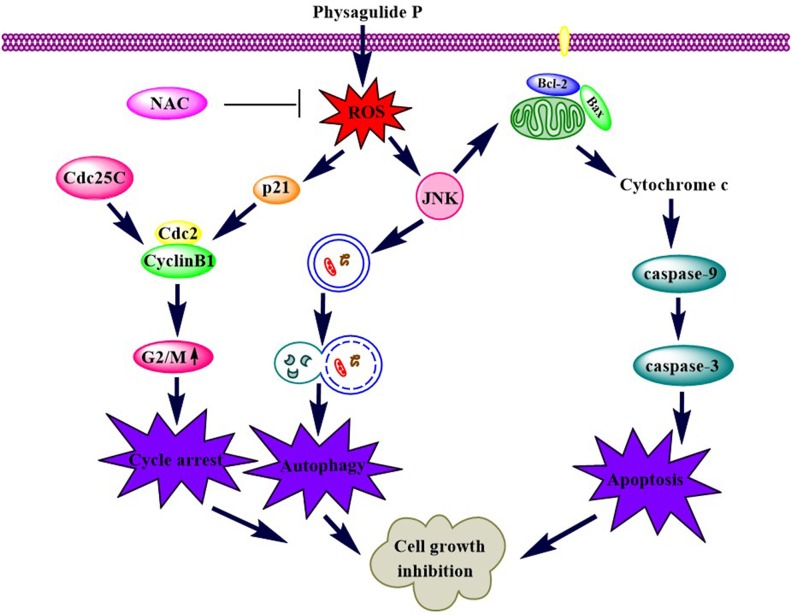
A schematic diagram of the pathways by which PP inhibits cell growth in triple-negative breast cancer

Our study showed that PP suppresses cell proliferation in triple-negative breast cancer cells by causing G2/M phase arrest, apoptosis and autophagy. In addition, PP induced apoptosis and autophagy by activating ROS/JNK signaling. Moreover, inhibiting autophagy with LC3B siRNA attenuated PP-induced cell growth inhibition, indicating that PP-induced autophagy is an anti-proliferative process in cells (Figure 10). These findings revealed multiple cytotoxic mechanisms for PP and demonstrated its potential for the treatment of triple-negative breast cancer cells.

## MATERIALS AND METHODS

### Cell culture and reagents

The triple-negative breast cancer cell lines MDA-MB-231 and MDA-MB-468 were purchased from the Cell Bank of the Shanghai Institute of Biochemistry and Cell Biology at the Chinese Academy of Sciences (Shanghai, China). MDA-MB-231and MDA-MB-468 were cultured in DMEM and L-15 media, respectively. The media were supplemented with 10% fetal bovine serum, 50 μg/ml penicillin and 50 μg/ml streptomycin at 37°C and 5% CO_2_ in a humidified environment.

PP was isolated from *Physalis angulata L*. at our laboratory, and samples containing at least 95% PP were used in all of our experiments (Supplementary Figure 1). PP was dissolved in dimethyl sulfoxide (DMSO) to obtain a stock solution of 50 mM and stored at 4°C. Before each experiment, the stock solution of PP was diluted with medium to different working concentrations, and control groups were treated with the same amount of DMSO (< 0.1%) in the corresponding experiments. Antibodies against Cyclin B1, Cdc2, Phospho-Cdc2 (Tyr15), Cdc25C (5H9, rabbit mAb), Phospho-Cdc25C (Ser216, rabbit mAb), p21, cleaved caspase-9 (Asp175, rabbit mAb), caspase-9, cleaved caspase-7 (Asp198), caspase-7, cleaved caspase-3 (Asp330), caspase-3, cleaved PARP (Asp214), PARP, LC3B (rabbit mAb), p62/SQSTM1 (rabbit mAb), Bax (D2E11, rabbit mAb), Bcl-2 (D55G8, rabbit mAb), phospho-SAPK/JNK (Thr183/Thr185, rabbit mAb), and SAPK/JNK (rabbit mAb) were purchased from Cell Signaling Technology (Danvers, MA, USA). β-Actin was purchased from Vazyme (Nanjing, China).

### Cell viability and tumor colony forming assays

MDA-MB-231 and MDA-MB-468 cells were treated with various concentrations of PP or 0.1% DMSO. Cell viability and tumor colony formation assays were performed as previously described [49].

### CFDA-SE cell tracer kit

MDA-MB-231 and MDA-MB-468 cells were labeled with CFDA-SE, plated in a 6-well plate and incubated overnight. The cells were then treated with different concentrations PP for 24 h before being harvested and washed twice with PBS. The fluorescence intensity was then measured with a BD Accuri C6 flow cytometer (Becton & Dickinson Co.).

### EdU incorporation assay

MDA-MB-231 and MDA-MB-468 cells were treated with various concentrations of PP or 0.1% DMSO. The EdU incorporation assay was performed using an EdU labeling/detection kit (Ribobio, Guangzhou, China). The operating methods were performed as previously described [53]. The cells were subsequently observed with an imageXpress^®^ confocal microscope (Molecular Devices, USA).

### Annexin V-alexa fluor 647/PI staining assay for cell apoptosis

MDA-MB-231 and MDA-MB-468 cells that underwent apoptosis were evaluated via flow cytometry. Briefly, MDA-MB-231 and MDA-MB-468 cells were treated with various concentrations of PP or 0.1% DMSO for 24 h. For flow cytometry, 1 × 10^6^ cells in 250 μl of binding buffer were stained with 5 μl of Annexin V-Alexa Fluor 647 and 10 μl of PI at room temperature in the dark for 15 min. The cells were then analyzed via flow cytometry (488 nm excitation and 647 nm emission filters) using BD Accuri C6 flow cytometer.

### Cell cycle analysis

In brief, after treatment with different concentrations of PP, the cells were harvested and fixed with 70% ethanol at −20°C overnight. The cells were then stained with PI/RNase Staining Buffer (Beyotime, Shanghai, China) for 15 min and analyzed with a BD Accuri C6 flow cytometer.

### Measurement of ROS

To measure the intracellular ROS levels, MDA-MB-231 and MDA-MB-468 cells were pretreated with various concentrations of PP or 0.1% DMSO for 24 h and then incubated in the dark with 10 mM of the oxidation-sensitive fluorescent probe 2′7′-dichlorfluorescein-diacetate (DCFH-DA, Beyotime) for 20 min at 37°C. DCFH-DA was cleaved by intracellular esterase to liberate free DCFH, and the ROS level was measured using a BD Accuri C6 flow cytometer.

### Measurement of the Δ*Ψ_m_*

Δ*Ψ_m_* was assessed with a JC-1 assay kit (KeyGen Biotech, Nanjing, China) as previously described [54]. Mitochondrial depolarization was measured by a decrease in the red/green fluorescence intensity ratio.

### Acridine orange (AO) staining

MDA-MB-231 and MDA-MB-468 cells were seeded in a 6-well plate and incubated with various concentrations of PP for 24 h. The cells were then harvested and stained with AO for 15 min. After washing with PBS, the cells were analyzed with a BD Accuri C6 flow cytometer.

### LC3B immunofluorescence

MDA-MB-231 and MDA-MB-468 cells were plated in 96-well culture plates at a density of 2 × 10^4^ cells per well and treated with different concentrations of PP for 18 h. The cells were then washed with PBS containing 0.05% saponin, fixed with 4% paraformaldehyde for 30 min, incubated with 0.5% Triton X-100 for 10 min and blocked with 5% BSA for 1 h. The cells were then incubated with antibodies against LC3B (Cell Signaling Technology, 1:50) overnight at 4°C; the nuclei were stained with Hoechst 33258 (Yeasen Biotechnology, Shanghai, China), and lysosomes were stained with Lysotracker for 30 min before imaging. Finally, images were taken with an imageXpress^®^ confocal microscope.

### Ad-mCherry-GFP-LC3B transfection

Cells were grown on 96-well plates until they reached 60%–70% confluence and then transfected with Ad-mCherry-GFP-LC3B adenovirus (Beyotime) at an MOI of 30 for 6 h at 37°C. Following treatment with PP for 18 h, the cells were stained with Hoechst 33342 (Yeasen). Finally, images were taken with an imageXpress^®^ confocal microscope.

### Western blotting analysis

MDA-MB-231 and MDA-MB-468 cells were lysed in western blotting lysis buffer and centrifuged at 12000 rpm for 10 min. Proteins were extracted for western blot as previously described [55]. Bound immuno-complexes were detected with a ChemiDOC^TM^ (Bio-Rad Laboratories, Hercules, CA).

### Gene knockdown using small interfering RNA

JNK siRNA [56], LC3B siRNA [57], p21 siRNA pools (siRNA1: CAGGCGGUUAUGAAAUUCAdTdT, siRNA2: GAUGGAACUUCGACUUUGUdTdT and siRNA3: CCUCUGGCAUUAGAAUUAUdTdT) and non-targeting siRNA were synthesized by Biomics (Nantong, China). For siRNA transfection, MDA-MB-231 cells were seeded in 6-well plates or glass-bottom dishes (1 × 10^6^ cells per well). SiRNAs (100 nmol/l) were introduced into the cells using electroporation according to the manufacturer's recommendations. The cells were then exposed to medium containing various concentrations of PP and harvested for further experiments.

### Cyt c immunofluorescence

MDA-MB-231 and MDA-MB-468 cells growing in glass-bottom dishes were treated with different concentrations of PP for 24 h. The cells were then fixed in 4% paraformaldehyde, washed with PBS, incubated with 0.5% Triton X-100 and blocked with 5% BSA. The cells were then incubated with primary antibodies against Cyt c (Cell Signaling Technology, 1:200) overnight at 4°C, followed by incubation with Alexa Flour 488-conjugated secondary antibody (Yeasen) for 2 h at room temperature in the dark. The nuclei were stained with Hoechst 33258, and lysosomes were stained with Mitotracker for 30 min before imaging. A laser scanning confocal microscope LSM 700 was used to analyze co-localization (LSM700, Carl Zeiss, Germany).

### Statistical analysis

All experiments were conducted more than three times. The results were analyzed using a one-way or two-way ANOVA followed by Tukey's range test. The data are presented as the mean ± standard error, and significance was set to *p* < 0.05.

## SUPPLEMENTARY MATERIALS FIGURE



## Abbreviations

PP: physagulide P
ROS: reactive oxygen species
JNK: c-Jun N-terminal kinases
ER: estrogen receptor
PR: progesterone receptor
CDKs: cyclin-dependent kinases
PCD: programmed cell death
SAPK: stress-activated protein kinase
AVOs: acidic vesicular organelles
CFDA-SE: carboxyfluorescein diacetate, succinimidyl ester
DMSO: dimethyl sulfoxide
MTT: 3-[4, 5-dimethylthiazole-2-yl]-2, 5-diphenyltetrazolium bromide
EdU: 5-ethynyl-20-deoxyuridine
PI: propidium iodide
DCFH-DA: 2′7′-dichlorfluorescein-diacetate
Δ*Ψ_m_*: mitochondrial membrane potential
AO: acridine orange
