# Supplementary material for: Anti-proliferation of triple-negative breast cancer cells with physagulide P: ROS/JNK signaling pathway induces apoptosis and autophagic cell death
Source: Oncotarget. 2017 Jul 17;8(38):64032–49. doi: 10.18632/oncotarget.19299 (PMC5609982; doi:10.18632/oncotarget.19299)
Supplement: Supplementary file 1 [file oncotarget-08-64032-s001.pdf]

# Anti-proliferation of triple-negative breast cancer cells with physagulide P: ROS/JNK signaling pathway induces apoptosis and autophagic cell death

## SUPPLEMENTARY FIGURE

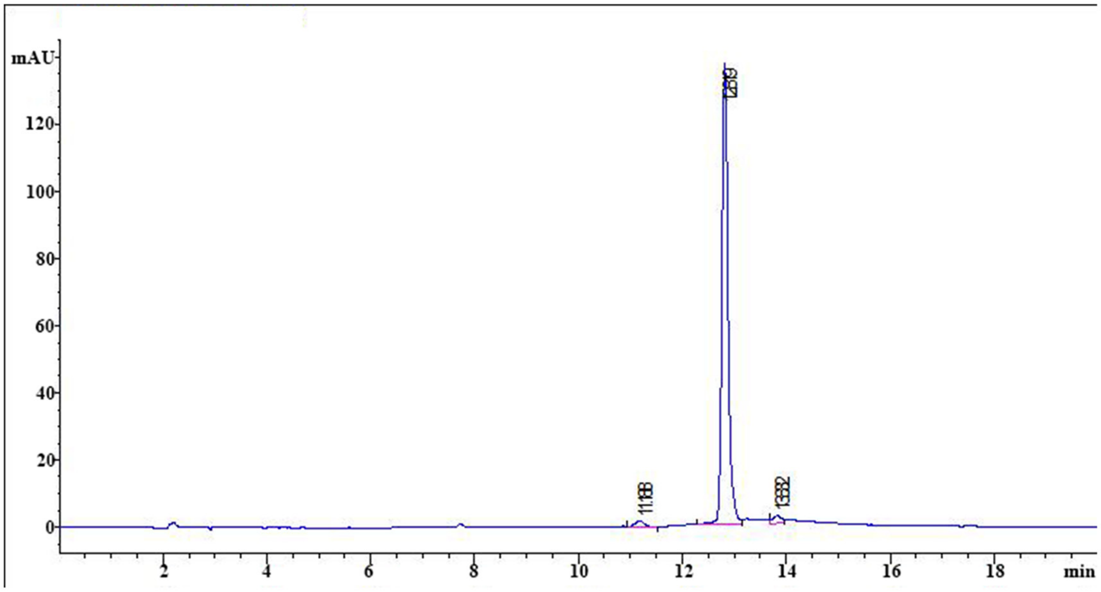


| Retention time | Area | Height | Width | Symmetry | Area% |
|---|---|---|---|---|---|
| 11.188 | 201.4 | 17.2 | 0.1842 | 1.16 | 1.697 |
| 12.819 | 111404.3 | 1372.8 | 0.1269 | 0.825 | 96.090 |
| 13.832 | 262.6 | 20.8 | 0.1792 | 1.027 | 2.213 |

**Figure 1: The HPLC of Physagulide P showed that the purity was 96.090% in solvent system of 50%-100% MeOH/ $H_2O$ and the retention time was 12.819 min (total time: 20min).**
